# Phylogeography of *Cranoglanis* (Teleostei: *Cranoglanididae*) Reveals Discordance Between Nominal Species and Maternal Lineages: A Broadly Distributed Clade Co-Occurring with a Cryptic Endemic in the Pearl River

**DOI:** 10.3390/ani16111648

**Published:** 2026-05-28

**Authors:** Xing-Pu Huang, Yan-Qiao Li, Tong Wu, Qi Huang, Ling-Lin Wan, Shao-Lin Xie, Ji-Xing Zou, Gui-Feng Wei, Qun Zhang

**Affiliations:** 1 Department of Ecology and Institute of Hydrobiology, Jinan University, Guangzhou 510632, China; 18933794228@163.com (X.-P.H.); yanqiao.liii@outlook.com (Y.-Q.L.); yisumonky@foxmail.com (T.W.); 15521302416@163.com (Q.H.); linglinwan@jnu.edu.cn (L.-L.W.); tweigf@jnu.edu.cn (G.-F.W.); 2 College of Marine Sciences, South China Agricultural University, Guangzhou 510642, China; xieshaolin@scau.edu.cn (S.-L.X.); zoujixing@scau.edu.cn (J.-X.Z.)

**Keywords:** *Cranoglanis*, mitochondrial cytochrome b gene, genetic diversity, lineage divergence, species conservation

## Abstract

The species limits of *Cranoglanis* remain controversial despite their relevance to freshwater fish conservation in southern China. We analyzed mitochondrial cytochrome b sequences from 203 individuals representing seven geographic populations across the Pearl, Red, and Nandujiang drainages to test species validity, assess genetic structure, and infer demographic history. Phylogenetic and haplotype network analyses recovered two deeply divergent lineages: a widespread Clade A distributed across all three drainages and a geographically restricted Clade B confined to the middle Xijiang system of the Pearl River Basin. The traditionally recognized Pearl River, Hainan, and Red River nominal taxa all fell within Clade A rather than forming three independent maternal lineages. Within Clade A, differentiation between the Yuanjiang–Red River and Pearl River populations was low, whereas the Nandujiang population showed significant differentiation and extremely low nucleotide diversity. Divergence dating estimated separation between Clades A and B at 4.9183 Ma. Both lineages showed signals consistent with historical demographic expansion. Our results support recognition of two evolutionarily significant units within *Cranoglanis* and identify the Nandujiang population as a priority for conservation, while the taxonomic status of Clade B requires further testing with nuclear and morphological evidence.

## Simple Summary

*Cranoglanis* catfishes are freshwater fishes native to the Pearl River, Red River, and Nandujiang River systems of southern China and northern Vietnam. Their classification has long been debated because forms from the Pearl River, Hainan Island, and the Red River have been treated as separate species. Using mitochondrial cytochrome b sequences from 203 individuals sampled across the full distribution range, we chose this marker because it is widely used in freshwater fish phylogeography and provides sufficient variation to distinguish both intraspecific and closely related interspecific lineages. We found that these named forms do not correspond to three separate maternal lineages. Instead, *Cranoglanis* contains two strongly divergent lineages: one is widespread across all three drainages, whereas the other is restricted to part of the Xijiang system in the Pearl River Basin and may represent a hidden, previously unrecognized taxon. We also found that the Nandujiang population on Hainan Island has extremely low genetic diversity, indicating elevated conservation concern. These results provide new molecular evidence for resolving the taxonomy of *Cranoglanis* and show that conservation should consider both the widespread lineage and the localized Xijiang lineage separately.

## 1. Introduction

Species are the fundamental units of biodiversity research and conservation practice, and accurate delimitation of species boundaries is essential for understanding biodiversity patterns and evolutionary history and for developing effective conservation strategies [1,2]. However, traditional morphology-based taxonomy has inherent limitations in detecting cryptic diversity, and taxa with highly conserved morphology often lead to an underestimation of true species diversity [3]. Because freshwater fishes are strongly constrained by drainage systems and generally have limited dispersal ability, they often exhibit a pronounced phylogeographic structure closely associated with a river network configuration, making them ideal systems for revealing cryptic diversity and testing species validity [4].

The genus *Cranoglanis*, belonging to the order *Siluriformes* and the family *Cranoglanididae*, comprises freshwater benthic fishes endemic to southern China, including the Pearl River, the Nandujiang River on Hainan Island, and the middle and upper reaches of the Red River, as well as northern Vietnam (Red River drainage) [5]. The validity of species within *Cranoglanis* has long been debated in the literature [6,7,8,9,10]. Chu et al. [5] treated *Cranoglanis* as a monotypic genus represented by *C. bouderius*, with two subspecies, *C. bouderius multiradiatus* from Hainan and *C. bouderius* from the Pearl River. By contrast, Ng et al. [11] recognized three morphologically defined species according to geographic origin, namely *C. multiradiatus* from Hainan, *C. bouderius* from the Pearl River, and *C. henrici* from the Red River, a treatment that has largely been adopted in current taxonomy. In contrast, Liu [12], Cheng et al. [13,14], and Xie et al. [15] argued that only a single valid species, *C. bouderius*, should be recognized within the genus because diagnostic morphological differences among the putative species or subspecies are limited, may largely reflect environmental effects [8], and are not accompanied by clear molecular lineage structures [9,10,11].

In recent years, natural habitats of *Cranoglanis* have continued to contract under multiple pressures, including human disturbance and biological invasions, and wild resources have declined sharply, placing their germplasm resources under severe threat [16,17]. As a result, *Cranoglanis* has been listed as vulnerable (VU) in the *China Red Data Book of Endangered Animals* [18], and its declining population trend has been well documented in both the Hainan and mainland populations [15]. To date, most genetic studies of *Cranoglanis* have focused on restricted geographic areas, such as local waters on Hainan Island or comparisons between Pearl River and Hainan populations [13,14,19,20], and therefore have not covered the full natural distribution of the genus. Only Xie et al. [15] analyzed *Cranoglanis* populations from the Yuanjiang, Pearl, and Hainan drainages using *cyt b* and *COI* sequences; however, their sampling coverage in the Xijiang system of the Pearl River Basin was limited and therefore insufficient to capture the actual genetic structure of this region. As the largest river system within the Pearl River Basin, the Xijiang River traverses diverse karst landscapes characterized by extensive subterranean rivers, complex hydrological networks, and substantial natural geographic isolation [21]. Located at the intersection of the karst region and the monsoonal climatic zone of southern China, this area has experienced frequent geological activity and climatic fluctuations. Such complex geological history and Quaternary climatic oscillations are widely regarded as major drivers of population divergence and the formation of cryptic diversity in freshwater fishes [22,23]. Nevertheless, no study based on comprehensive sampling of representative geographic populations across the full distribution range of *Cranoglanis* has yet explicitly tested its species boundaries, and its evolutionary history therefore remains unclear.

Phylogeography provides an effective framework for integrating population genetic structure with historical biogeographic inference [4]. The mitochondrial cytochrome b (*cyt b*) gene has been widely used in freshwater fish species delimitation and phylogeographic studies because it provides sufficient variation at both the intraspecific and closely related interspecific levels while showing relatively limited saturation [24,25]. The *cyt b* gene has been widely used in freshwater fish phylogeography since the 1990s, initially for tracing postglacial recolonization in temperate species [26] and later extended to Asian freshwater fishes to test species boundaries and assess tectonic and climatic drivers of lineage divergence [27,28]. Based on *cyt b* sequences from 203 individuals representing seven geographic populations across the entire distribution range of *Cranoglanis*, the present study aimed to (1) reconstruct the maternal phylogenetic relationships within *Cranoglanis* and test whether the three currently recognized nominal species each correspond to an independent maternal lineage unit; (2) assess the degree of genetic differentiation among geographic populations and identify evolutionarily significant units (ESUs); (3) estimate divergence times among the major lineages and explore the mechanisms driving lineage divergence in the context of regional geology and paleoclimate; and (4) evaluate the conservation priority of geographic populations in order to provide a genetic basis for the conservation and management of *Cranoglanis*.

## 2. Materials and Methods

### 2.1. Sample Collection and Preservation

**Figure 1:**
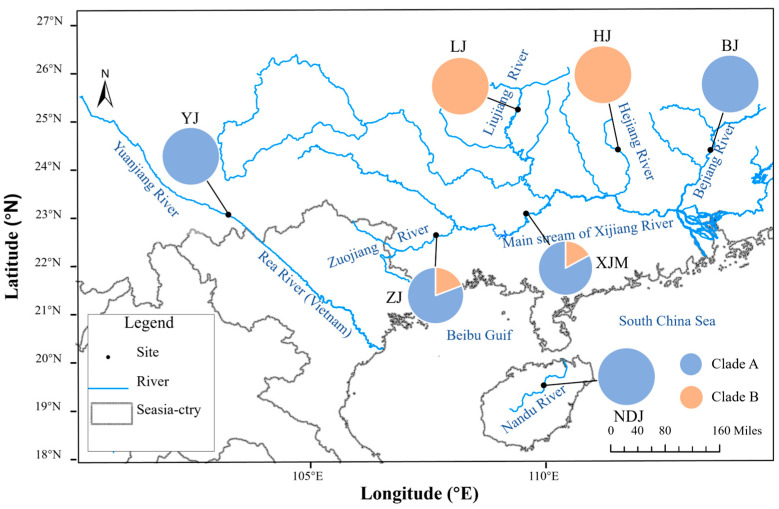
Map of the study area. Sampling localities and mitochondrial *cyt b* lineage composition of *Cranoglanis* in the Yuanjiang River, Pearl River and Nandujiang River basins. Pie charts at each site show the proportional frequencies of genetic lineages, with colors denoting different haplotypes as indicated in the figure legend. Site codes (YJ, LJ, HJ, ZJ, XJM, BJ, and NDJ) correspond to those in other figures.

Between February 2022 and November 2025, a total of 203 specimens representing seven geographic populations from three major drainage systems, namely the Red River, Pearl River, and Nandujiang River, were collected across the entire natural distribution range of *Cranoglanis* (Figure 1; Table 1). All samples were obtained from catches of local fishermen using gill nets, cast nets, and trap nets. Specimens were morphologically identified with reference to *Fauna Sinica: Osteichthyes*, *Siluriformes* [5] and then preserved in 95% ethanol. All 203 individuals were successfully amplified and sequenced for the mitochondrial *cyt b* gene and included in the downstream population genetic and phylogenetic analyses.

### 2.2. Genomic DNA Extraction, Amplification, and Sequencing

Genomic DNA was extracted using a saturated phenol–chloroform extraction protocol [29]. The *cyt b* primers were synthesized by Guangzhou Tianyi Huiyuan Gene Technology Co., Ltd. (Guangzhou, China). PCR amplification was carried out in a total reaction volume of 20 μL, containing 10 μL of the Taq PCR Master Mix, 7 μL of ddH_2_O, 1 μL of the forward primer, 1 μL of the reverse primer, and 1 μL of template DNA. The Taq PCR Master Mix was purchased from Guangzhou Tianyi Huiyuan Gene Technology Co., Ltd. (Guangzhou, China). The PCR cycling conditions were as follows: an initial denaturation at 94 °C for 5 min and 30 cycles of 94 °C for 30 s, 55 °C for 30 s, and 72 °C for 1 min, followed by a final extension at 72 °C for 10 min. PCR products were examined by 1% agarose gel electrophoresis, and qualified amplicons were subjected to bidirectional Sanger sequencing by Guangzhou Tianyi Huiyuan Gene Technology Co., Ltd. (Guangzhou, China).

### 2.3. Data Analysis

#### 2.3.1. Sequence Processing and Estimation of Genetic Diversity

Raw sequencing data were manually edited and multiple sequence alignment was performed using Bioedit 7.0.9.0 [30] and Chromas software. Nucleotide composition, base variation values, and transition and transversion values were calculated, and the number of mutation sites was counted in MEGA 7.0 [31]. Pairwise Kimura 2-parameter (K2P) distances were also computed in MEGA 7.0 and exported as a distance matrix. Principal coordinate analysis (PCoA) of this matrix was subsequently performed using the ape package (v5.8.1) in R 4.6.0 to visualize genetic relationships among individuals. The number of haplotypes, haplotype diversity (Hd) and nucleotide diversity (π) [32] were calculated, and mismatch distribution analysis was performed using DnaSP 6.12.03 [33]. A haplotype network was constructed using PopART 1.7 [34].

#### 2.3.2. Phylogenetic Reconstruction and Divergence Time Estimation

A time-calibrated Bayesian phylogenetic tree was reconstructed in BEAST v2.7.7 (see Section 2.3.4 for detailed parameters). The resulting maximum clade credibility (MCC) tree was visualized in FigTree v1.4.4. Clade A and Clade B were defined by reciprocal monophyly and a large mutational gap (36 fixed differences) in phylogenetic and network analyses (see Section 3.2). All downstream population genetic analyses were therefore conducted separately for each clade to avoid inter-lineage divergence artificially inflating overall differentiation and obscuring lineage-specific demographic history.

#### 2.3.3. Population Genetic Structure Analysis

Pairwise genetic differentiation was assessed using the fixation index (*F*_ST_) in Arlequin 3.5.2.2 [35,36]. The distribution of genetic variation was further evaluated by analysis of molecular variance (AMOVA) [37], and statistical significance was tested using 1000 permutations. Spatial analysis of molecular variance was performed in SAMOVA 2.0 for K = 2–6. The smallest K value that produced a relatively high among-group component (*F*_CT_) together with geographically coherent groups (K = 4) was selected as an additional grouping scheme for AMOVA. MIGRATE 3.7.2 was used to estimate θ (effective population size) and M (migration rate), and gene flow was calculated as *N*_m_ = θ × M/x [38], where x is a fixed coefficient and was set to 1 for mitochondrial markers [39]. Gene flow patterns were visualized in R 4.6.0 using the circlize (v0.4.18) and tidyverse (v2.0.0) packages.

#### 2.3.4. Historical Demography Analysis

Neutrality tests were performed by calculating Tajima’s D [40] and Fu’s FS [41], together with the sum of squared deviations (SSD) [42], Harpending’s raggedness index (Rg), and the expansion parameter τ [43]. Population expansion time was estimated using the formula T = (τ/2μk) × generation time, where T is the time since expansion, τ is the expansion parameter, μ is the mutation rate per sequence and generation, and k is the sequence length. The mutation rate of *cyt b* was assumed to be 2% per million years [44,45].

BEAST v2.7.7 [46] was used for divergence time estimation, with Ictalurus punctatus as the outgroup. Following Rabosky et al. [47], the divergence between *Cranoglanis* and Ictalurus punctatus was calibrated at 59.4 Ma and used as the calibration point for the local molecular clock analysis of *Cranoglanis*. A strict clock was used because the in-group showed limited divergence; the HKY model was selected via jModelTest 2. This fossil-based calibration derives from a robust siluriform phylogeny, and uncertainty in the estimated divergence is expressed as the 95% HPD interval. Markov chain Monte Carlo (MCMC) analyses [48] were run for 100 million generations, with sampling every 2000 generations. The first 10% of samples were discarded as burn-in. Convergence and sampling adequacy were assessed in Tracer v1.7.2, where effective sample size (ESS) values were all well above 200, indicating adequate sampling and good convergence. A maximum clade credibility (MCC) tree was generated using TreeAnnotator and visualized in FigTree v1.4.4. Bayesian skyline plots (BSPs) were reconstructed in BEAST v2.7.7 under the HKY model, a strict molecular clock, and the Coalescent Bayesian Skyline model [49,50,51], and the results were visualized in Tracer v1.7.2.

#### 2.3.5. Ancestral Area Reconstruction

Ancestral area reconstruction was performed in RASP using the S-DIVA model, based on the MCC tree, to infer dispersal and vicariance events [52,53].

## 3. Results

### 3.1. Sequence Characteristics and Genetic Diversity of Cranoglanis cyt b

**Figure 2:**
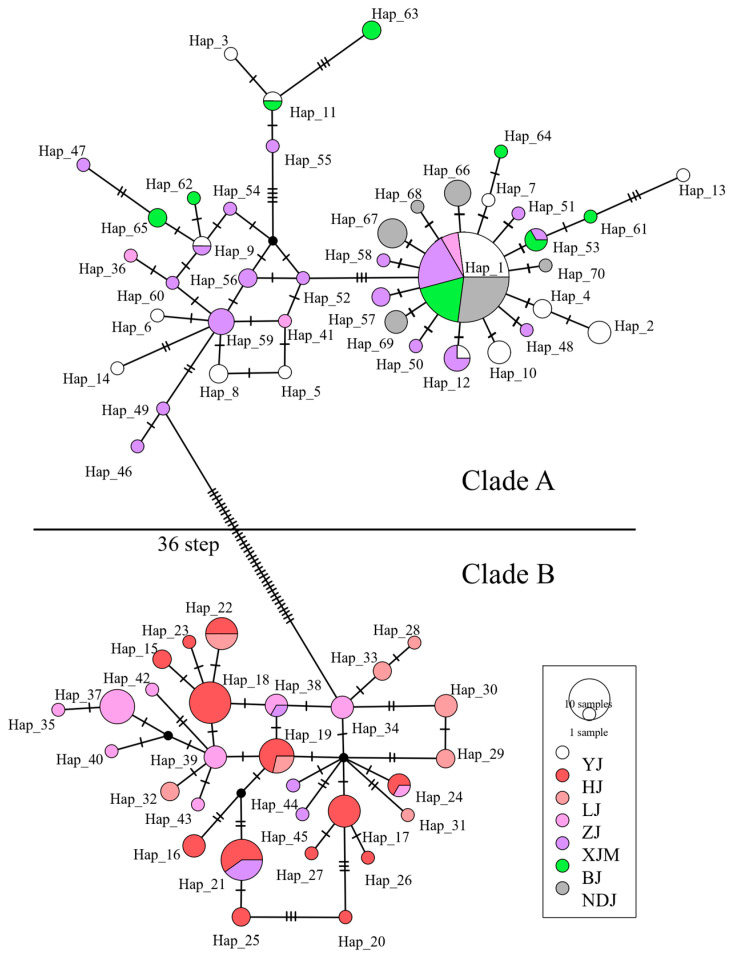
Haplotype network constructed based on the mitochondrial *Cyt b* gene of *Cranoglanis.* Note: Each pie chart represents a haplotype, with different patterns indicating different geographic groups. The numbers inside the patterns represent haplotype types. Black dots represent theoretically existing but extinct or undiscovered haplotypes. The circle area reflects haplotype frequency (quantity). The small lines on connecting lines between circles and pie charts represent single mutation steps.

A total of 203 mitochondrial *cyt b* gene sequences were obtained from seven populations, and the aligned sequence length was 1138 bp. Phylogenetic (MCC tree) and network analyses (Figure 2) identified two reciprocally monophyletic, deeply divergent maternal lineages—Clade A and Clade B—separated by 36 fixed nucleotide substitutions and supported by a Bayesian posterior probability of 1.0 (Figure S1). Because this deep split dominates the among-individual genetic variation, all diversity, differentiation, and demographic analyses were performed both globally and separately for each clade, thereby avoiding the confounding effects of inter-lineage divergence on estimates of within-lineage patterns. Genetic diversity results are summarized in Table 2. At the overall level, *Cranoglanis* showed high haplotype diversity (Hd = 0.934 ± 0.013) and high nucleotide diversity (π = 0.01967 ± 0.00041). This elevated overall π reflects the deep divergence between the two clades and does not represent within-lineage diversity. Lineage-specific analyses showed that Hd and π were 0.827 ± 0.036 and 0.00300 ± 0.00029 in Clade A and 0.949 ± 0.009 and 0.00337 ± 0.00016 in Clade B, respectively. In both lineages, π values were below 0.005, indicating comparatively low nucleotide diversity.

Among the geographic populations within Clade A, haplotype diversity was highest in the YJ population (Hd = 0.827 ± 0.063) and lowest in the ZJ population (Hd = 0.700 ± 0.218). Nucleotide diversity was highest in the BJ population (π = 0.00378 ± 0.00075) and lowest in the NDJ population (π = 0.00082 ± 0.00013). The latter value was extremely low, accounting for only approximately one quarter of the mean value of the other populations, suggesting substantially reduced genetic diversity in the Nandujiang population. Within Clade B, haplotype diversity was highest in the LJ population (Hd = 0.917 ± 0.037) and lowest in the XJM population (Hd = 0.714 ± 0.181). For nucleotide diversity, the highest value was observed in the LJ population (π = 0.00324 ± 0.00024), whereas the lowest was detected in the ZJ population (π = 0.00242 ± 0.00025).

### 3.2. Genetic Differentiation Among Cranoglanis Populations and Phylogenetic Relationships Among Haplotypes

A total of 95 variable sites were detected, defining 70 haplotypes. Among them, haplotype Hap1 was represented by 48 individuals, Hap18 and Hap21 by 10 individuals each, Hap37 by seven individuals, and Hap17 by six individuals, whereas most of the remaining haplotypes were represented by only 1–5 individuals. In terms of haplotype distribution, no shared haplotypes were observed among populations except for Hap1. Hap1 was shared among some populations from the Yuanjiang–Red River, Pearl River, and Nandujiang River basins, whereas the remaining haplotypes were mainly private to individual basins.

As shown in Figure 2, the haplotype network clearly separated *Cranoglanis* haplotypes into two genetic lineages, Clade A and Clade B, which were separated by 36 mutational steps and exhibited marked geographic clustering. The YJ, BJ, and NDJ populations were found exclusively within Clade A, whereas the LJ and HJ populations occurred only within Clade B. By contrast, the XJM and ZJ populations were distributed across both lineages. Within Clade A, Hap1 formed a typical secondary center with a star-like structure and was connected to multiple low-frequency haplotypes.

### 3.3. Population Genetic Structure of Cranoglanis

The genetic differentiation index (*F*_ST_) among the seven geographic populations ranged from 0.002 to 0.978, indicating highly uneven levels of differentiation among population pairs, with most comparisons reaching statistical significance. The overall extremely high *F*_ST_ values largely reflected the combined signal of the two lineages. To better assess the actual levels of genetic differentiation within each lineage, Clades A and B were analyzed separately.

FST analyses (Table S2) confirmed weak differentiation within Clade A, with only NDJ showing moderate isolation (FST = 0.107–0.164), whereas all Clade B populations were significantly differentiated from each other (FST = 0.093–0.372).

Dxy and Da further supported this genetic structure (Table 3). Within Clade A, Dxy was consistently low (0.002–0.004) and Da approached zero, with NDJ exhibiting only marginally higher net divergence from other populations. Within Clade B, Dxy (0.003–0.004) and Da (≤0.001) mirrored the significant FST-based differentiation, indicating fine-scale geographic isolation among the Xijiang tributary populations. Between Clades A and B, Dxy was 0.038 and Da was 0.035, values that exceed typical intraspecific thresholds and support the inference of deep lineage divergence.

**Figure 3:**
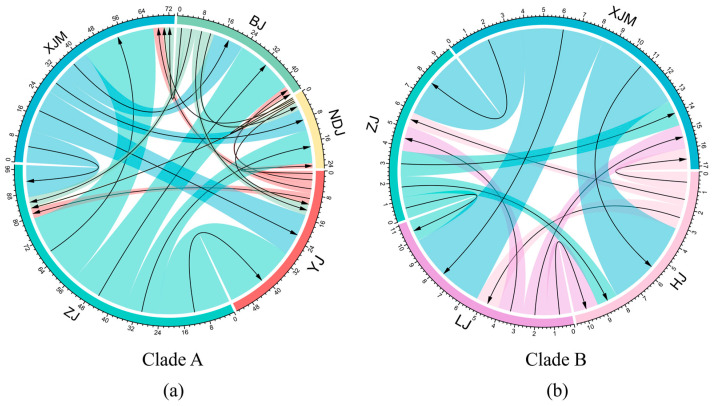
(**a**) Gene flow of Clade A in *Cranoglanis*. (**b**) Gene flow of Clade B in *Cranoglanis*.

Principal coordinate analysis (PCoA) of the K2P distance matrix clearly separated all individuals into two non-overlapping clusters corresponding to Clades A and B (Figure 3). The first axis explained 98.3% of the total variation, reflecting the deep divergence between the clades. Within Clade A, individuals from different drainages intermingled without distinct geographic clustering, whereas Clade B formed a tighter cluster. The PCoA results corroborate the deep lineage separation and limited geographic structure observed in the haplotype network and phylogenetic analyses.

AMOVA results are presented in Table 4. Under the first grouping scheme, most of the genetic variation was attributed to differences among populations (91.66%), far exceeding the proportion within populations (8.34%), with a highly significant overall *F*_ST_ of 0.91658. Under the second grouping scheme, based on the three conventional drainage basins, 25.95% of the variation occurred among groups, 66.70% among populations within groups, and 7.35% within populations, with a highly significant *F*_SC_ of 0.90080. These results suggest that the genetic structure of *Cranoglanis* is not explained simply by the traditional drainage basin framework, but rather reflects strong geographic isolation within the Pearl River Basin. Under the third grouping scheme, based on the optimal SAMOVA solution, the proportion of variation among groups increased to 78.52%, whereas that among populations within groups decreased to 14.00%, and that within populations was 7.48%. Although the corresponding *F*_CT_ value (0.78519) was not statistically significant, it was markedly higher than that under the conventional drainage-based grouping and therefore better captured the actual genetic structure of *Cranoglanis*. The non-significant FCT likely reflects limited statistical power from small within-group sample sizes rather than a lack of biological differentiation. Overall, across all three grouping schemes, most of the genetic variation was partitioned among populations and among groups, whereas variation within populations remained low, indicating restricted gene flow among *Cranoglanis* populations and a strong influence of intrabasin geographic isolation. Traditional drainage divisions therefore do not appear to be the principal factor shaping the observed genetic structure.

Bidirectional gene flow among populations within Clades A and B, estimated using MIGRATE 3.7.2, is shown in Figure 3. In Clade A (Figure 3a), relatively high levels of gene flow were detected among BJ, YJ, ZJ, and XJM, suggesting relatively frequent genetic exchange. Among these, BJ and YJ mainly acted as recipients of gene flow, whereas XJM and ZJ contributed more as sources, accounting for 28.73% and 53.74% of the total outward gene flow, respectively. By contrast, the NDJ population received most of the inferred gene flow and contributed almost no outward gene flow. In Clade B (Figure 3b), LJ, HJ, and ZJ tended to function mainly as recipients of gene flow, whereas XJM contributed a greater proportion of outward gene flow, accounting for 54.77% of the total.

### 3.4. Historical Demography of Cranoglanis

**Figure 4:**
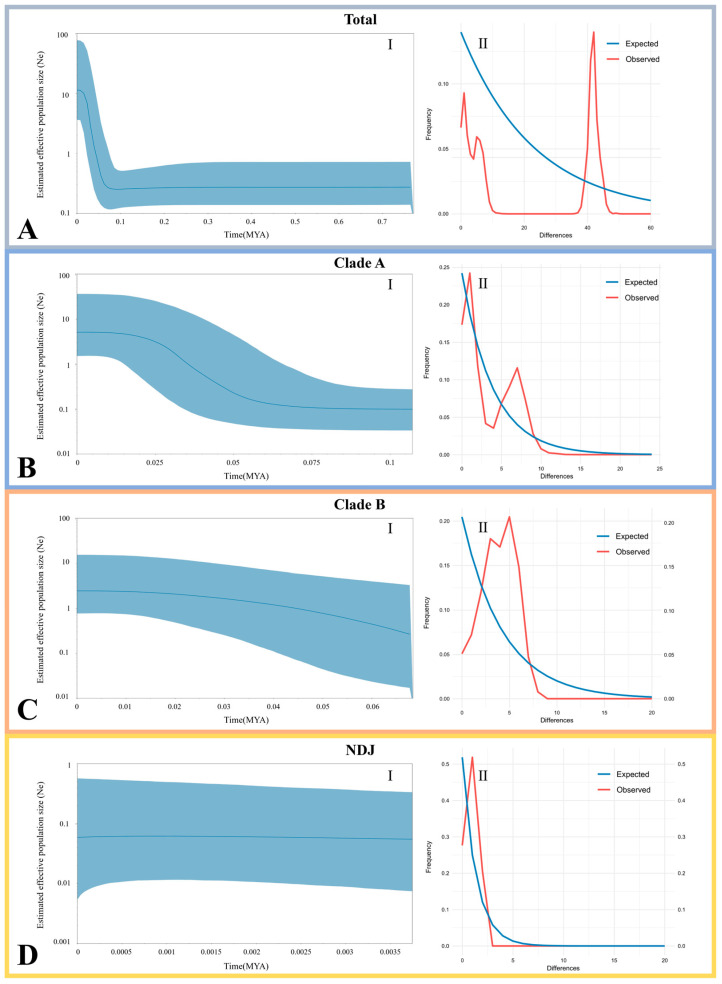
Bayesian skyline plots (BSPs) and nucleotide mismatch distribution of *Cranoglanis*. (**A**) total, (**B**) Clade A, (**C**): Clade B, and (**D**) NDJ. In each panel, I = BPS and II = nucleotide mismatch distribution. For the BSPs, the blue lines show the median estimates of the effective population size (*N*_e_) through time, with the shaded areas representing the 95% highest posterior density (HPD) intervals around these estimates.

The overall neutrality test results for *Cranoglanis* are shown in Table 2. Tajima’s D was 1.196. Fu’s FS was −6.607, and τ was 37.780. At the overall level, Tajima’s D was positive but not significant, whereas Fu’s FS was negative but not significant. Both SSD and Rg were low and non-significant (SSD = 0.033; Rg = 0.015). In contrast to the neutrality test results, the overall mismatch distribution of *Cranoglanis* showed a clearly bimodal pattern (Figure 4(AII)), with major peaks at 0–10 and 38–46 pairwise differences, whereas frequencies were close to zero across the interval of 11–36 differences. This pattern reflected the combined signal of the two lineages.

To reduce the confounding effect of lineage admixture on interpretation of the overall analysis, Clades A and B were analyzed separately. The results showed that Tajima’s D values for both Clade A and Clade B were negative but not significant (−1.711 and −1.176, respectively), whereas Fu’s FS values were highly significantly negative (−26.135 and −14.355, respectively). In addition, both SSD and Rg were low and non-significant (SSD = 0.023, Rg = 0.033). The significantly negative Fu’s FS values and Bayesian skyline plots (Figure 4(BI,CI)) support recent demographic expansions in Clades A and B. The multimodal mismatch distributions, however, do not conform to a simple panmictic expansion model; this pattern likely reflects the internal population substructure within each clade, which masks the expansion signal in mismatch analyses.

At the lineage level, Clade A showed a multimodal mismatch distribution (Figure 4(BII)), which was not fully consistent with its highly significantly negative Fu’s FS value and non-significant SSD (SSD = 0.005). This pattern may indicate the presence of multiple substructures within Clade A, thereby masking the expansion signal inferred from the neutrality tests. The Bayesian skyline plot further supported a recent expansion trend in Clade A (Figure 4(BI)). By contrast, the NDJ population exhibited a mismatch distribution strongly concentrated near zero pairwise differences (Figure 4(DII)), which was inconsistent with its highly significant SSD value. This pattern may reflect stochastic effects of a small effective population size, highly limited gene flow, and genetic drift, leading to a simplified multimodal distribution. The Bayesian skyline plot further indicated that the NDJ population did not experience recent demographic expansion (Figure 4(DI)); thus, the NDJ population exhibits reduced genetic variation and a stable or shrinking population size. Clade B showed a weakly multimodal mismatch distribution (Figure 4(CII)), which was also inconsistent with the neutrality test results. Clade B showed a similar pattern, consistent with internal substructure rather than simple expansion.

### 3.5. Divergence Time Between Cranoglanis Lineages and Ancestral Area Reconstruction

The maximum clade credibility (MCC) tree showed that the 70 haplotypes were divided into Clade A and Clade B (Figure S1), with a Bayesian posterior probability of one at the node separating the two clades, indicating very strong support. Based on molecular clock analysis, the divergence time between Clade A and Clade B was estimated at 4.9183 Ma, with a 95% highest posterior density (HPD) interval for node heights of 3.3854–6.6138 Ma.

S-DIVA analysis reconstructed the vicariance events and geographic distribution history of different *Cranoglanis* populations. A total of nine high-confidence events with probabilities > 0.5 were identified (Table 5). The results indicated a complex historical biogeographic pattern in *Cranoglanis* and suggested that XJM may have been the primary center of dispersal origin, whereas HJ and ZJ may have served as secondary centers receiving dispersal from XJM.

## 4. Discussion

### 4.1. Species Validity

The TCS haplotype network indicated that *Cranoglanis* comprises two markedly divergent evolutionarily significant units, namely the widespread Clade A, which occurs across the three major drainage basins, and Clade B, which is restricted to the Xijiang system of the Pearl River Basin. The non-overlapping PCoA clustering further supports the proposition that Clades A and B represent evolutionarily independent units. Although multiple divergent lineages occurring within a single drainage are not uncommon in freshwater fishes, such cases appear to be relatively rare within the Xijiang system. Only a few comparable examples have been reported, such as the similar pattern of divergence and distribution observed between *Ptychidio jordani* and *P. longibarbus* in the genus *Ptychidio* reported by Zheng et al. [28].

Pairwise nucleotide divergence (Dxy) between Clade A and Clade B was 0.038, and net divergence (Da) was 0.035, values that exceed typical intraspecific *cyt b* thresholds in freshwater fish (0.02) [54]. Within-clade Dxy values were 0.002–0.004, and principal coordinate analysis (PCoA) showed two completely non-overlapping clusters corresponding exactly to these clades (Figure 3). This deep mitochondrial divergence, together with the absence of gene flow in sympatry, supports the interpretation that the three previously recognized Pearl River, Hainan, and Red River nominal taxa all belong to a single species within Clade A, which is consistent with the conclusions by Xie et al. [15] and Liu [12].

The deep divergence between Clades A and B and the absence of shared haplotypes argue against recent introgression or sex-biased dispersal. Thus, the *cyt b* data suggest that the three nominal forms belong to a single biological species, although nuclear markers are required to exclude alternative explanations.

By contrast, Clade B may represent an undescribed cryptic species. Comparable levels of mtDNA divergence have been used to distinguish cryptic species in other freshwater fish [55], illustrating the utility of mitochondrial markers for generating species-level hypotheses. However, because this inference is based solely on a maternally inherited locus, formal recognition of Clade B as a distinct species requires independent evidence from nuclear markers and morphology.

### 4.2. Genetic Structure and Genetic Diversity

From the perspective of genetic patterning, both lineages showed low levels of nucleotide diversity, which may indicate some limitation in their long-term adaptive potential and thus imply potential conservation concern [56]. The lineage-specific nucleotide diversities of Clade A (π = 0.00300) and Clade B (π = 0.00337) are within the typical range reported for freshwater fish, while the overall high diversity of *Cranoglanis* (π = 0.01967) is driven by the deep divergence between the two clades; a similar pattern of high total nucleotide diversity reflecting cryptic lineage structure has been observed in other widely distributed species in the region, including *Hemiculter leucisculus* (π = 0.02900) [57] and *Silurus asotus* (π = 0.01799) [58] (Table S1). In Clade A, the NDJ population showed significant genetic differentiation from all other populations. This pattern is likely associated with postglacial sea-level rise, which isolated Hainan Island from the mainland by the Qiongzhou Strait and thereby reduced connectivity between the Nandujiang population and continental populations. Correspondingly, inward gene flow accounted for the vast majority of inferred migration involving NDJ, whereas outward gene flow was nearly absent, and the population exhibited extremely low nucleotide diversity (π = 0.00082), which is among the lowest recorded for Chinese freshwater fishes and is comparable to that of the endangered catfish *Hemibagrus guttatus* (π = 0.00042; Table S1) [59], further emphasizing its vulnerable status. Together, these results suggest marked genetic isolation and possible genetic homogenization in the Hainan population, indicating that this population warrants elevated priority in future conservation monitoring and management. Similar links between strong isolation, reduced genetic diversity, and elevated conservation concern have been reported in other freshwater fish studies. For example, comparably low genetic diversity and significant differentiation from mainland populations have been documented in insular freshwater fishes on Hainan Island, such as *Barbodes semifasciolatus* [60] and *Opsariichthys hainanensis* [61], all of which are isolated in the Qiongzhou Strait and face similar conservation challenges.

By contrast, the *F*_ST_ values between the Yuanjiang–Red River and Pearl River populations were clearly lower than those between either of these regions and the Nandujiang population. This pattern suggests that the geographic isolation between the Yuanjiang–Red River and Pearl River systems may have occurred more recently than the separation between Hainan Island and the mainland. That interpretation is broadly consistent with phylogeographic studies from South China showing that island–mainland separation and subsequent hydrological isolation can leave stronger genetic signatures than more recent or less complete isolation among mainland drainages.

Because only one sampling site was included per river, within-drainage genetic variation may be underestimated, which could inflate apparent differentiation among populations and lead to conservation units that appear more isolated than they truly are. Future multi-site sampling is therefore necessary to validate the generality of these results.

### 4.3. Historical Demography

The demographic history of *Cranoglanis* further reflects the influence of geological and climatic changes on genetic divergence in freshwater fishes. The maximum clade credibility (MCC) tree suggested that Clades A and B diverged at approximately 4.9 Ma, a period that may have coincided with substantial tectonic and geomorphic reorganization in southern China [62]. Such landscape changes could have promoted isolation among river systems, and the subsequent onset of the Quaternary glaciations at about 2.58–2.6 Ma may have further intensified habitat fragmentation in *Cranoglanis* [63], ultimately facilitating the independent evolution of Clades A and B [64].

The τ values indicated that the principal expansion phases of Clades A and B occurred at approximately 574 ka and 471 ka, respectively, corresponding to the Mindel–Riss interglacial period [65]. Climatic warming and enhanced hydrological connectivity during this interval may have promoted rapid dispersal in *Cranoglanis* [66,67,68]. The relatively higher haplotype diversity of Clade A may further suggest that it represents the earlier expanding lineage. Comparable phylogeographic patterns have been documented in other freshwater fishes of southern China: for instance, *Hemiculter leucisculus* shows lineage divergence at ∼4.5 Ma and demographic expansion during the mid-Pleistocene interglacials [69]; *Toxabramis houdemeri* exhibits signals of recent expansion following Pleistocene climatic amelioration [44].

Bayesian skyline plot analyses indicated that the most recent expansion in both lineages occurred at around 60 ka, during Marine Isotope Stage 3 (MIS 3), when East Asia is widely considered to have experienced relatively warm and humid conditions together with intensified monsoonal influence. Under such conditions, increased precipitation may have restored river connectivity and created favorable opportunities for dispersal in *Cranoglanis* [70,71,72].

The S-DIVA results and bidirectional gene flow patterns in the Xijiang system suggested that this region may have acted as a center of dispersal for *Cranoglanis* populations, a pattern broadly consistent with phylogeographic inferences reported for *Hemiculter leucisculus* [57,69]. From this perspective, the previously recognized Hainan and Red River forms are better interpreted as belonging to the same species-level lineage as the Pearl River taxon rather than representing distinct species. The Xijiang drainage has a complex hydrological network, occupies a geographically central position among the three major drainage regions considered here, and includes extensive subterranean river systems. In addition, seasonal flooding in southern China may have intermittently connected adjacent drainages, thereby creating opportunities for cross-basin dispersal and gene flow in *Cranoglanis*. Based on the phylogenetic topology, Clades A and B were most likely derived from a common ancestral lineage.

As a benthic fish that prefers slow-flowing waters, *Cranoglanis* is likely to have limited capacity to traverse rapid currents and structurally complex shoals. The Liujiang and Hejiang rivers are located on the southwestern flank of the Nanling Mountains and are characterized by substantial elevational gradients between upstream and downstream sections. During Quaternary sea-level fall, intensified river incision may have progressively produced hydrological barriers such as gorges, rapids, and shoals [64,73,74,75], thereby restricting gene flow between the Liujiang and Hejiang populations and downstream populations and contributing to divergence between the two lineages. With subsequent climatic warming, at least partial reconnection among river systems may have allowed populations of Clade B to disperse into other drainages, eventually generating the present-day distribution pattern of the two *Cranoglanis* lineages. The S-DIVA results further suggested that the Hejiang River may have served as a major dispersal source area for Clade B.

### 4.4. Conservation Implications

Taken together, Clades A and B should be treated as two independent conservation units, and indiscriminate translocation across lineages should be avoided in order to prevent disruption of their natural genetic structure. Such caution is well founded: translocations across divergent genetic lineages have caused genetic homogenization and loss of local adaptation in several freshwater fishes, including *Salmo trutta* in Europe [76] and *dwarf galaxias* in Australia [77], reinforcing the principle that genetically distinct units should be managed separately. Given the marked differences between the two lineages in geographic distribution and genetic characteristics, conservation strategies should also differ accordingly. For the widely distributed Clade A, which shows relatively low internal genetic differentiation, maintaining ecological connectivity is important to prevent habitat fragmentation from further restricting natural gene flow. For the NDJ population, which exhibits extremely low nucleotide diversity, targeted conservation actions should be implemented, and any population reinforcement should be conducted cautiously and on a scientifically informed basis in order to restore genetic diversity in the wild population.

For the geographically restricted and more strongly differentiated Clade B, cross-drainage stocking should be avoided to reduce the risk of disrupting the native gene pool. In addition, for the ZJ and XJM populations where the two lineages co-occur, river connectivity should be maintained and anthropogenic habitat fragmentation should be minimized; all stocking activities should preferentially use source populations from within the same drainage to preserve the natural genetic pattern. As the Xijiang mainstream may represent a potential center of origin or dispersal for *Cranoglanis*, maintaining hydrological connectivity in this system may be particularly important, and anthropogenic barriers that impede dispersal should be minimized as far as possible.

## 5. Conclusions

Two deeply divergent evolutionarily significant units were identified: the widespread Clade A and the Xijiang-endemic Clade B. The previously recognized Pearl River, Hainan, and Red River forms were all assigned to Clade A, whereas Clade B may represent a putative cryptic species, although this interpretation remains tentative without independent evidence from nuclear markers and morphology. The divergence time between the two lineages was estimated at 4.9183 Ma. In addition, the *Cranoglanis* population from the Nandujiang River on Hainan Island exhibited extremely low nucleotide diversity, indicating that this extreme genetic impoverishment likely results from the population’s long-term isolation on Hainan Island and therefore requires elevated conservation attention. Overall, this study provides molecular evidence relevant to the long-standing taxonomic debate surrounding *Cranoglanis* and offers a scientific basis for its conservation and sustainable utilization. Future work should expand both sampling density and geographic coverage and incorporate complete mitochondrial genomes together with nuclear markers to improve the robustness of the inferences.

## Figures and Tables

**Table 1:** Sampling information of *Cranoglanis.*

Basin	System	Locality	Sample Size	Sampling Date
Yuanjiang River	Yuanjiang River (YJ)	Honghe, Yunnan Province, China	32	2025.11
Pearl River	Liujiang River (LJ)	Liuzhou, Guangxi Province, China	16	2022.3–2023.6
	Hejiang River (HJ)	Hezhou, Guangxi Province, China	43	2022.3–2023.6
	Zuojiang River (ZJ)	Chongzuo, Guangxi Province, China	25	2025.11
	Xijiang Mainstream (XJM)	Guigang, Guangxi Province, China	41	2025.11
	Beijiang River (BJ)	Shaoguan, Guangdong Province, China	19	2025.11
Nandujiang River	Nandujiang River (NDJ)	Danzhou Hainan Province, China	27	2022.2
Total			203	

**Table 2:** Genetic diversity: neutrality tests of *Cranoglanis.*

Site		s	h	Hd ± SD	π ± SD	Tajima’s D	Fu’s FS	τ
Basin	System							
Yuanjiang River	YJ	24	14	0.827 ± 0.063	0.00333 ± 0.00058	−1.275	−3.628	5.265
Pearl River	LJ	11	8	0.917 ± 0.037	0.00324 ± 0.00024	0.420	−0.856	4.568
	HJ	16	13	0.896 ± 0.024	0.00316 ± 0.00021	0.358	−1.648	4.285
	ZJ Clade A	7	3	0.700 ± 0.218	0.00316 ± 0.00104	0.498	1.674	5.582
	ZJ Clade B	10	9	0.853 ± 0.061	0.00242 ± 0.00025	−0.075	−2.055	3.627
	ZJ Total	50	12	0.897 ± 0.042	0.01368 ± 0.00332	0.676	2.914	38.554
	XJM Clade A	20	18	0.900 ± 0.040	0.00338 ± 0.00037	−0.724	−7.825 ***	3.200
	XJM Clade B	8	4	0.714 ± 0.181	0.00293 ± 0.00071	0.109	0.870	5.205
	XJM Total	60	22	0.924 ± 0.029	0.01322 ± 0.00266	0.262	−0.739	39.649
	BJ	17	8	0.772 ± 0.093	0.00378 ± 0.00075	0.130	0.130	6.990
Pearl River Clade A		27	26	0.851 ± 0.045	0.00348 ± 0.00035	−1.033	−13.728 ***	4.422
Pearl River Total		83	55	0.958 ± 0.008	0.01950 ± 0.00057	1.521	−3.380	37.452
Nandujiang River	NDJ	5	6	0.724 ± 0.071	0.00082 ± 0.00013	−0.797	−2.019	0.621
Clade A		41	41	0.827 ± 0.036	0.00300 ± 0.00029	−1.711	−26.135 ***	5.223
Clade B/Pearl River Clade B		31	29	0.949 ± 0.009	0.00337 ± 0.00016	−1.176	−14.355 ***	4.285
All		95	70	0.934 ± 0.013	0.01967 ± 0.00041	1.196	−6.607	37.780

Note: Significance levels are indicated by *** *p* < 0.001. For Tajima’s D and Fu’s FS, significant values (with asterisks) indicate departures from neutral mutation–drift equilibrium.

**Table 3:** Dxy and Da between populations of Clade A and Clade B in *Cranoglanis.*

Clade A											
		YJ	ZJ		XJM			BJ	PR Total		NDJ
YJ									**0.000**		**0.000**
ZJ						0.000		0.000			
XJM			0.003					0.000			
BJ			0.003			0.004					
PR Total		**0.0** **03**									**0.001**
NDJ		**0.** **002**							**0.** **003**		
Clade B											
	LJ			HJ			ZJ			XJM	
LJ				0.000			0.001			0.001	
HJ	0.004						0.001			0.000	
ZJ	0.004			0.003						0.001	
XJM	0.004			0.003			0.004				

Note: Abbreviations: PR, Pearl River Basin. Below diagonal: Dxy (average number of nucleotide differences per site between populations); above diagonal: Da (net nucleotide divergence between populations). For Clade A, values in bold indicate genetic differentiation between different river basins. Non-bold values indicate genetic differentiation among different river systems within the Pearl River Basin. Blank cells represent pairwise comparisons that were not computed independently, because the relevant populations were pooled into a larger group for hierarchical analysis.

**Table 4:** Molecular variance analysis results of the *Cranoglanis* population structure.

Grouping Basis	Item	Among Groups	Among Populations Within Groups	Within Populations	Total	*F* Statistics (*p*-Value)
7 Geographical Populations	d.f	6	N/A	196	202	
	Sum of squares	859.883	N/A	89.491	949.37	*F*_ST_: 0.91658 ***
	Variance components	5.01672	N/A	0.45659	5.47331	
	Percentage of variation	91.66	N/A	8.34		
Yuanjiang River Group, Pearl River Group, and Nandujiang River Group	d.f	2	4	196	202	
	Sum of squares	398.418	461.465	89.491	949.374	*F*_SC_: 0.90080 ***
	Variance components	1.61312	4.14608	0.45659	6.21578	*F*_ST_: 0.92654 ***
	Percentage of variation	25.95	66.70	7.35		*F*_CT_: 0.25952
Group 1 (YJ), Group 2 (HJ), Group 3 (ZJ,XJM), and Group 4 (LJ,BJ,NDJ)	d.f	3	3	196	202	
	Sum of squares	797.571	62.311	89.491	949.374	*F*_SC_: 0.65165 ***
	Variance components	4.79082	0.85411	0.45659	6.10152	*F*_ST_: 0.92517 ***
	Percentage of variation	78.52	14.00	7.48		*F*_CT_: 0.78519

Note: Results are shown for three hierarchical grouping schemes: (i) all seven geographic populations analyzed without higher-level grouping; (ii) populations grouped by drainage basin (Yuanjiang group (including YJ), Pearl River group (including ZJ, HJ, LJ, and XJM), and Nandujiang group (including NDJ)); and (iii) four groups defined by the best SAMOVA solution: (Group 1 (including YJ), Group 2 (including HJ), Group 3 (including ZJ and XJM) and Group 4 (including LJ, BJ, and NDJ)). Significance levels are indicated by *** *p* < 0.001.

**Table 5:** High-confidence dispersal and vicariance events identified by S-DIVA analysis for *Cranoglanis.*

Event Route	Probability
X→YX	1.0000
X→XB	1.0000
H→HL	1.0000
H→HX	1.0000
Z→LZ	0.9474
X→YX→Y|X	0.7500
X→ZX	0.7481
X→YZXBN	0.6376
Z→HZ	0.6270

Note: Vicariance events with probability > 0.5 are considered high-confidence events. Area code: B = BJ, X = XJM, H = HJ, L = LJ, Y = YJ, Z = ZJ. α and β represent pre-defined discrete geographic areas of *Cranoglanis*; αβ = combined distribution area in α and β; α|β = vicariance split of the original area into two independent subpopulations (α and β).

## Data Availability

The mitochondrial haplotype sequences of *Cranoglanis* generated in this study were deposited in the NCBI GenBank database under accession numbers PZ348032—PZ348101. The remaining data supporting the findings of this study are included in this article.

## Supplementary Materials

The following supporting information can be downloaded at https://www.mdpi.com/article/10.3390/ani16111648/s1, Figure S1: MCC analysis of *Cranoglanis*; Table S1: Comparison of genetic diversity indices based on the mitochondrial cyt b gene between *Cranoglanis* and sympatric freshwater fishes; Table S2: *F*_st_ between populations of Clade A and Clade B in *Cranoglanis*; Figure S2: Principal Coordinates Analysis (PCoA) of *Cranoglanis*. References [57,58,59] are cited in the Supplementary Materials.

## Abbreviations

The following abbreviations are used in this manuscript:

YJ	Yuanjiang River
LJ	Liujiang River
HJ	Hejiang River
ZJ	Zuojiang River
XJM	Xijiang Mainstream
BJ	Beijiang River
NDJ	Nandujiang River
*Cyt b*	*Cytochrome b*
Hd	Haplotype Diversity
π	Nucleotide Diversity
AMOVA	Analysis of Molecular Variance
*F*_ST_	Fixation Index (Genetic Differentiation Coefficient)
*N*_m_	Gene Flow Among Populations
SSD	Sum of Squared Deviations
Rg	Harpending’s Raggedness Index
BSPs	Bayesian Skyline Plots
MCC	Maximum Clade Credibility
