# Supplementary material for: Phylogeography of Cranoglanis (Teleostei: Cranoglanididae) Reveals Discordance Between Nominal Species and Maternal Lineages: A Broadly Distributed Clade Co-Occurring with a Cryptic Endemic in the Pearl River
Source: Animals (Basel). 2026 May 28;16(11):1648. doi: 10.3390/ani16111648 (PMC13255799; doi:10.3390/ani16111648)
Supplement: Supplementary file 1 [file animals-16-01648-s001.zip › animals-4324139-supplementary.pdf]

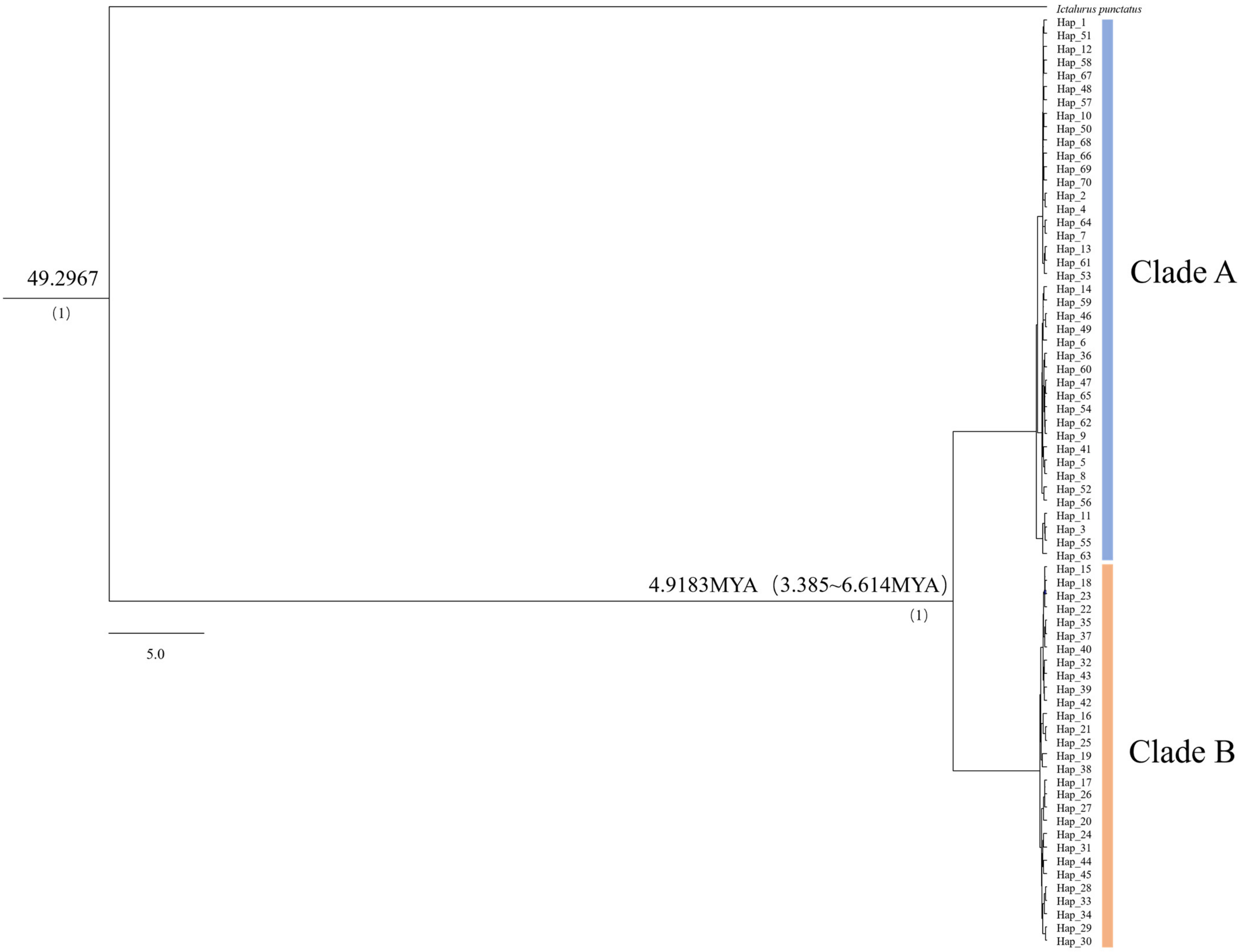

Figure S1 MCC analysis of *Cranoglanis*. Divergence times are shown above branches with 95% highest posterior density (HPD) intervals in parentheses; Bayesian posterior probabilities are indicated below branches. The scale bar represents divergence time (Ma).

Table S1 Comparison of genetic diversity indices based on the mitochondrial cyt b gene between Cranoglanis and sympatric freshwater fishes

Species	Region	Conservation status	Hd±SD	π ± SD	Source
Cranoglanis (Clade A)	Southern China	VU(China Red Data Book)	0.827 ± 0.036	0.00300 ± 0.00029	This study
Cranoglanis (Clade B)	Pearl River	VU(China Red Data Book)	0.949 ± 0.009	0.00337 ± 0.00016	This study
Cranoglanis (NDJ)	Hainan island	VU(China Red Data Book)	0.724 ± 0.071	0.00082 ± 0.00013	This study
Hemiculter leucisculus	Southern China	Least Concern (IUCN)	0.973 ± 0.004	0.02900 ± 0.00130	Gu et al.[57]
Silurus asotus	China	Least Concern (IUCN)	0.948 ± 0.009	0.01799 ± 0.00005	Xu et al.[58]
Hemibagrus guttatus	Pearl River	Protected (Class II, China, wild only)	0.465 ± 0.054	0.00042 ± 0.00007	Kuang et al.[59]

Note: VU = Vulnerable; IUCN = International Union for Conservation of Nature. *Hemibagrus guttatus* is listed as a National Second-Class Protected Wild Animal in China, but this protection applies exclusively to wild populations; artificially bred individuals are not covered.

Table S2 F_{st} between populations of Clade A and Clade B in *Cranoglanis*

Clade A						
	YJ	ZJ	XJM	BJ	PR Total	NDJ
YJ						
ZJ						
XJM		-0.091				
BJ		-0.048	0.016			
PR Total	**0.017**					
NDJ	**0.164*****				**0.107*****	

Clade B				
	LJ	HJ	ZJ	XJM
LJ				
HJ	0.120***			
ZJ	0.219***	0.154***		
XJM	0.271***	0.093	0.372***	

Note: Significance levels are indicated by ***P<0.001. Abbreviations: PR, Pearl River Basin. For Clade A, values in bold indicate genetic differentiation between different river basins. Non-bold values indicate genetic differentiation among different river systems within the Pearl River Basin. Blank cells represent pairwise comparisons that were not computed independently, because the relevant populations were pooled into a larger group for hierarchical analysis.

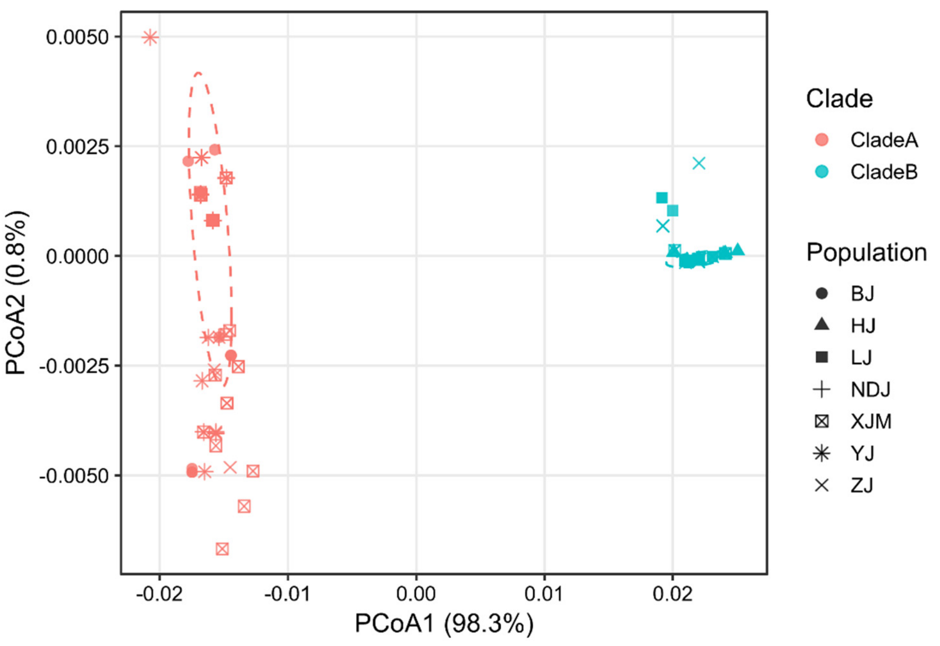

Figure S2 Principal Coordinates Analysis (PCoA) of *Cranoglanis* .
